# A Cerato-Platanin Family Protein FocCP1 Is Essential for the Penetration and Virulence of *Fusarium oxysporum* f. sp. *cubense* Tropical Race 4

**DOI:** 10.3390/ijms20153785

**Published:** 2019-08-02

**Authors:** Siwen Liu, Bo Wu, Jing Yang, Fangcheng Bi, Tao Dong, Qiaosong Yang, Chunhua Hu, Dandan Xiang, Hongrui Chen, Huoqing Huang, Chuange Shao, Yixiang Chen, Ganjun Yi, Chunyu Li, Xiuwu Guo

**Affiliations:** 1 College of Horticulture, Shenyang Agricultural University, Shenyang 110866, China; 2 Key Laboratory of South Subtropical Fruit Biology and Genetic Resource Utilization, Ministry of Agriculture, Key laboratory of Tropical and Subtropical Fruit Tree Research of Guangdong Province, Institution of Fruit Tree Research, Guangdong Academy of Agricultural Sciences, Guangzhou 510640, China

**Keywords:** *Fusarium oxysporum* f. sp. *cubense*, cerato-platanin, virulence, necrosis

## Abstract

*Fusarium oxysporum* f. sp. *cubense* tropical race 4 (*Foc* TR4) is well-known as the causal agent of *Fusarium* wilt of banana and is one of the most destructive phytopathogens for banana plants. The molecular mechanisms underlying *Foc* TR4 virulence remain elusive. Here, we demonstrate that a cerato-platanin (CP) protein, FocCP1, functions as a virulence factor that is required by *Foc* TR4 for penetration and full virulence. The *FocCP1* gene was expressed in every condition studied, showing a high transcript level in planta at the early stage of infection. Infiltration of the recombinant FocCP1 protein induced significant cell death and upregulated defence-related gene expression. *FocCP1* knock-out strains showed a significant decrease in aerial growth rather than aqueous growth, which is reminiscent of hydrophobins. Furthermore, deletion of *FocCP1* significantly reduced virulence and dramatically reduced infective growth in banana roots, likely resulting from a defective penetration ability. Taken together, the results of this study provide novel insight into the function of the recently identified *FocCP1* as a virulence factor in *Foc* TR4.

## 1. Introduction

*Fusarium oxysporum* f. sp. *cubense* (*Foc*), a soil-borne hyphomycete, causes the most destructive disease to bananas. This disease has reduced the production of bananas in Asia, Africa, Australia, and the tropical Americas over several decades [1,2,3]. Four races of *Foc* have been recognized thus far based on the banana cultivars that can be infected [4,5]. *Foc* tropical race 4 (TR4) is highly virulent toward almost all banana cultivars and has now been detected outside Southeast Asia, namely in the Middle East and Mozambique [1,6,7]. Despite the serious damage caused by *Foc* TR4, efficient strategies for its management are not available to date and the understanding of its pathogenesis is still rudimentary [8].

Plants have evolved the ability to detect pathogen-associated molecular patterns (PAMPs), which can be recognized by receptors on the surface of plant cells that activate PAMP-triggered immunity (PTI) [9]. Fungi have also evolved ways to overcome plant basal defences by secreting effectors. However, plants in turn have evolved to combat these effectors with the help of resistance proteins that activate effector-triggered immunity (ETI), such as the hypersensitive response (HR), which is a form of programmed cell death [9,10,11]. To further understand the banana–*Foc* interaction, proteins that are required for virulence, either PAMPs or effectors, must be identified. In *F*. *oxysporum* f. sp. *lycopersici* (*Fol*), the “secreted in xylem” (*SIX*) genes have been identified as a family of effectors [12]. All *SIX* gene homologues in *Foc* TR4 have been identified by taking advantage of a whole-genome comparison approach, but only *SIX1a* has been studied in terms of its role in the banana–*Foc* interaction [13]. Besides virulence-related proteins, several researchers focused on phytotoxins to elucidate the pathogenicity mechanisms of *Foc* TR4, such as beauvericin (BEA) and fusaric acid (FSA) [14,15]. FSA is considered to be involved in virulence in banana plants and can cause browning of vascular cells and plant necrosis [14]. FSA biosynthesis gene mutants of *Foc* TR4 have been shown to be defective in FSA production and exhibit reduced virulence toward banana plants, indicating that FSA is essential for the full virulence of *Foc* TR4 [15].

Transcriptome profiling of resistant and susceptible banana roots following inoculation with *Foc* TR4 was analyzed. Data not only yielded considerable knowledge about putative resistance genes in banana plants, but also provided a resource for predicting secreted *Foc* TR4 effector candidates (data not published). Among the effector candidates, several small secreted cysteine-rich proteins (SSCRP) have been hypothesized to be strong candidates for pathogenesis-related effectors. Among the SSCRPs identified, one interesting candidate, FOIG_10415, was identified as a homologous protein of cerato-platanin (CP) in *Foc* TR4 and was subsequently named FocCP1. The CP protein family was first described and found in *Ceratocystis fimbriata*, an ascomycete pathogenic to the European plane tree [16]. Since then, CP proteins have been reported in many different filamentous fungi and have also been identified as secreted proteins both in Ascomycota and Basidiomycota. Although CP proteins are secreted into the culture filtrate, they are also found in the cell wall of ascospores, conidia, and hyphae [17]. Recently, the CP protein Epl1 from *Trichoderma harzianum* was observed to be transported to the fungal cell wall, where it is probably involved in cell wall interactions with host cells [18]. CP proteins have been shown to act as virulence factors or elicitors (acting as PAMPs) of the plant defence response. For example, the CP-like protein BcSPl1 from *Botrytis cinerea* were proven to be elicitors at the appropriate concentration and the intensity of the defence response led to HR and cell death [19]. MgSM1 from *Magnaporthe oryzae* was shown to have potential in generating disease-resistant crops through a transgenic ectopic expression approach [20]. Meanwhile, the Sm1 and Epl1 proteins from *Trichoderma virens* and *Trichoderma atroviride* were shown to induce systemic resistance in host and non-host plants against different lifestyle pathogens [21,22]. Moreover, several other CP proteins, such as MSP1 from *Magnaporthe grisea*, CP-like protein from *Ceratocystis fimbriata,* and SsCP1 from *Sclerotinia sclerotiorum,* were shown to act as virulence factors [16,23,24]. Further study revealed that SsCP1 interacts directly with the pathogenesis-related protein PR1 in planta and contributes to the virulence of *S. sclerotiorum* [24]. Recently, a CP protein was characterized from the secretome of *Foc*, which was named FocCP1. It was shown that purified FocCP1 triggers HR and systemic acquired resistance (SAR) in tobacco, acting as an elicitor. In this study, we report FocCP1 in *Foc* TR4, which contributes to the penetration process and virulence, as a knock-out mutant, showing reduced growth in banana plants. Results from infiltration of a recombinant FocCP1 protein into banana leaves indicate that the FocCP1 protein may also function as a virulence factor in *Foc* TR4 pathogenesis. These findings not only improve our understanding of the molecular mechanism of *Foc* TR4 in disease development, but also provide an important experimental basis for virulence research in *Foc* TR4.

## 2. Results

### 2.1. Identification and Characterization of a CP1 Homologue in Foc TR4

FocCP1 is an SSCRP containing 140 amino acid residues with a CP domain (residues 19–138) and a signal peptide at the cleavage site between amino acids 18 and 19 (SignalP 4.1 server), suggesting that it may be a secreted protein. FocCP1 contains four cysteine residues (C39, C78, C81, and C136), which is typical of the CP family. In addition, hydropathicity plot (Kyte and Doolittle) analysis showed that FocCP1 contains a high percentage of hydrophobic residues (Figure S1, see supporting information).

**Figure 1:**
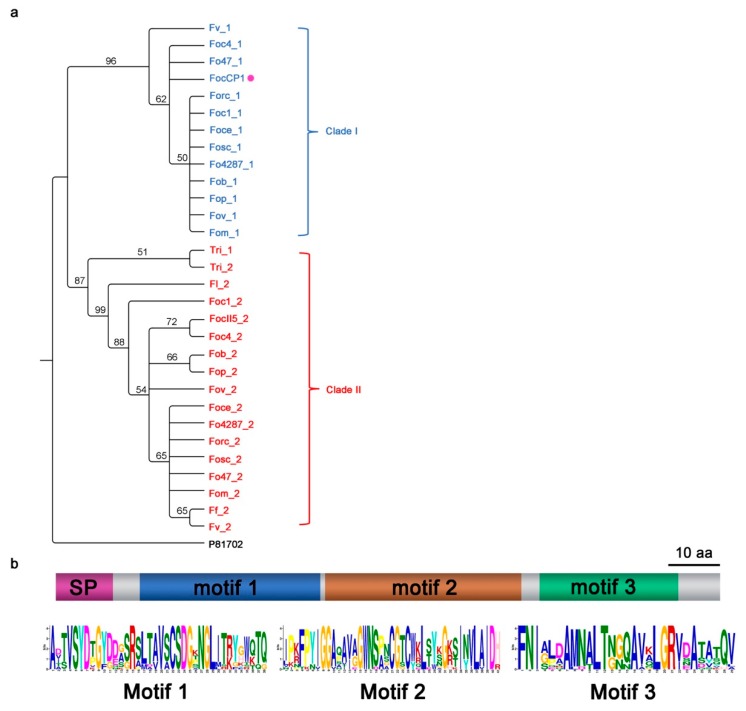
Analysis of the cerato-platanin (CP) protein FocCP1. (**a**) Maximum likelihood tree based on CP (P81702) and its 32 orthologous proteins from *Fusarium* spp. and *Trichoderma atroviride* genomes. The number of branches was the bootstrap support percentages of each branch and branches with <50% support rate were condensed. (**b**) Conserved motifs of CP proteins predicted with the MEME suite. All analyzed CP proteins exhibited three motifs.

Most fungal species have only one CP protein [25], but in *Fusarium* genomes, except for *Fusarium fujikuroi* (*Ff*) and *Fusarium langsethiae* (*Fl*)*,* two orthologues of CP were identified that clustered into two paralogous clades (Figure 1a). Except for *Ff* and *Fl* and the outgroup *Trichoderma atroviride* (*Tri*), in which no clade I protein was identified, a single clade I protein was present in each of the *Fusarium* genomes. *Tri* had two clade II proteins in its genome that were tandem repeats on the chromosomes. Although both clade I and clade II *Fusarium* CP genes were orthologues of P81702 (CP protein of *Ceratocystis fimbriata* f. sp. *platani*), clade I *FocCP1* shared more sequence similarity with P81702, suggesting they could have similar functions. In the CP domain, 26 amino acid residues were highly conserved in all the protein sequences, showing high sequence conservation of the two genes in the *Fusarium* species, indicating that they are functionally important (Figure S2). Searching for motifs by MEME indicated that these CP proteins may have three conserved motifs (Figure 1b).

### 2.2. FocCP1 Is Highly Expressed during Spore Germination and Infection Progress

**Figure 2:**
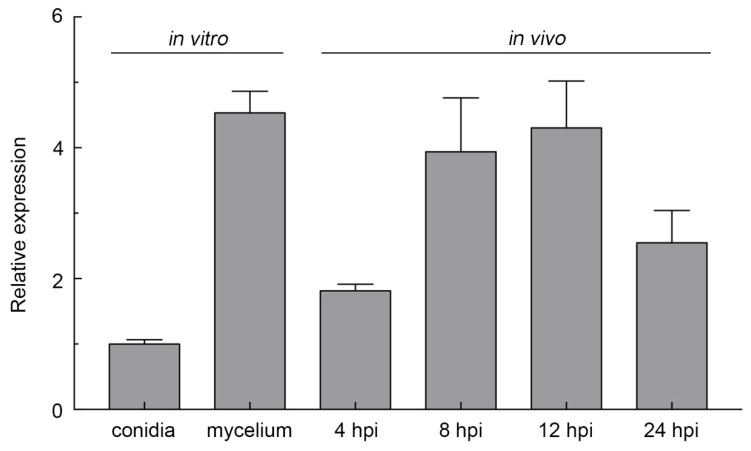
Transcript levels of FocCP1 at different developmental stages. Transcript levels were normalized to the expression of the fungal reference gene *EF1α*. Data are the means ± SDs from three independent experiments.

*FocCP1* was originally identified from early expression genes in a transcriptome analysis of *Foc* TR4-infected banana roots (data not published). Therefore, we investigated the expression levels of FocCP1 using qRT-PCR, including in vitro hyphal formation and infected roots at different time points of early infection stages. Our results revealed that the transcript levels of FocCP1 greatly increased during conidia germination and increased gradually during early infection progress but declined at 24 h post-inoculation (hpi) (Figure 2). As FocCP1 can be produced during conidia germination and hyphal growth continually, it appeared to be related to host penetration at early infection stages.

### 2.3. Generation of FocCP1-Mutated and FocCP1-Complemented Strains

**Figure 3:**
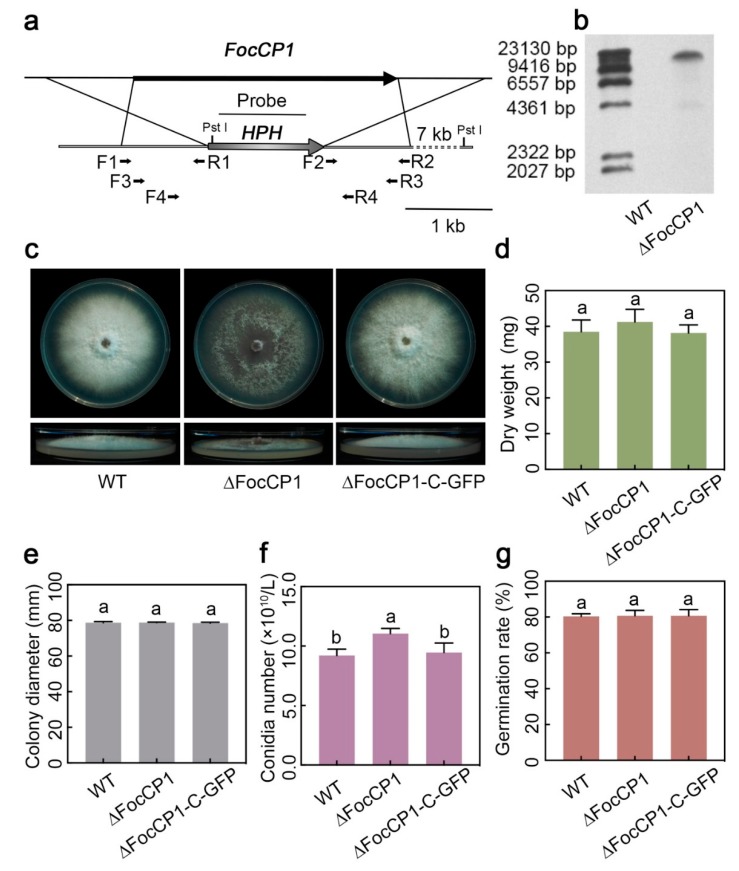
Characterization and vegetative growth of the ∆FocCP1 mutant strain and its complementary strain ∆FocCP1-C-GFP. (**a**) Schematic strategy used for generation of the targeted disruption strain ∆FocCP1; hygromycin-resistance cassette (*HPH*) fragments used as the probes for hybridization are indicated. (**b**) Southern blotting of the targeted disruption strain ∆FocCP1. (**c**) Colony morphologies of wild type (WT), ∆FocCP1, and ∆FocCP1-C-GFP on potato dextrose agar (PDA) plates. Photos were taken 6 days after incubation. (**d**) Dry weight of WT, ∆FocCP1, and ∆FocCP1-C-GFP. Hyphae were collected from 5-day cultures of ∆FocCP1 and ∆FocCP1-C-GFP strains grown in potato dextrose broth PDB. (**e**) Colony diameter (**f**) conidia production and (**g**) conidial germination of the WT, ∆FocCP1, and ∆FocCP1-C-GFP strains. Conidia were collected from 5-day cultures of each strain grown for 5 days in MLB. Conidia production was counted with a haemocytometer. Conidia were incubated in extract peptone dextrose (YEPD) broth for 12 h and the germination rate was determined by randomly collecting 100 conidia. Data presented in (**d**–**g**) are means ± SDs from three independent experiments. The letters above the histograms indicate significant differences at *p* < 0.05 between the WT and the ∆FocCP1 and ∆FocCP1-C-GFP strains (Student’s *t*-test).

To determine the function of the *FocCP1* gene, we generated a gene replacement construct containing a hygromycin-resistance gene cassette (*HPH*) and transformed it into the *Foc* TR4 reference strain II5 (Figure 3a). Candidate hygromycin-resistant transformants were screened by PCR with the primer pairs listed in Table S1. A deletion mutant was further confirmed by Southern hybridization assays using an *HPH*-specific probe (Figure 3b). We also generated complement strains by introducing FocCP1 fused with green fluorescent protein (GFP) at the carboxyl terminus under the native promoter into one representative deletion strain, ∆FocCP1-C-GFP.

No differences were observed among the ∆FocCP1, ∆FocCP1-C-GFP, and wild-type (WT) strains for radial or hyphal growth on potato dextrose agar (PDA) (Figure 3c,e), whereas the deletion mutant ∆FocCP1 exhibited a reduction in the formation of aerial hyphae on PDA (Figure 3c). To determine whether FocCP1 only influenced aerial growth but not aqueous growth, we further inoculated these strains into potato dextrose broth (PDB), where no difference in fungal biomass formation (dry weight) was found between the WT and the ∆FocCP1 strains (Figure 3d). When the same amount of conidia was inoculated in mung bean liquid (MBL) broth, the ∆FocCP1 strain produced even more conidia than the WT and complemented strains, resulting in an increase of 23% (Figure 3f). The conidia produced by the ∆FocCP1 strain were able to germinate with germination rates similar to those of the WT and complemented strains (Figure 3g). These results indicate that targeted disruption of FocCP1 increases conidiation, but has no influence on aqueous growth or conidia germination.

### 2.4. The FocCP1 Protein is Carried by Vacuoles and Predominantly Functions in the Apoplast

**Figure 4:**
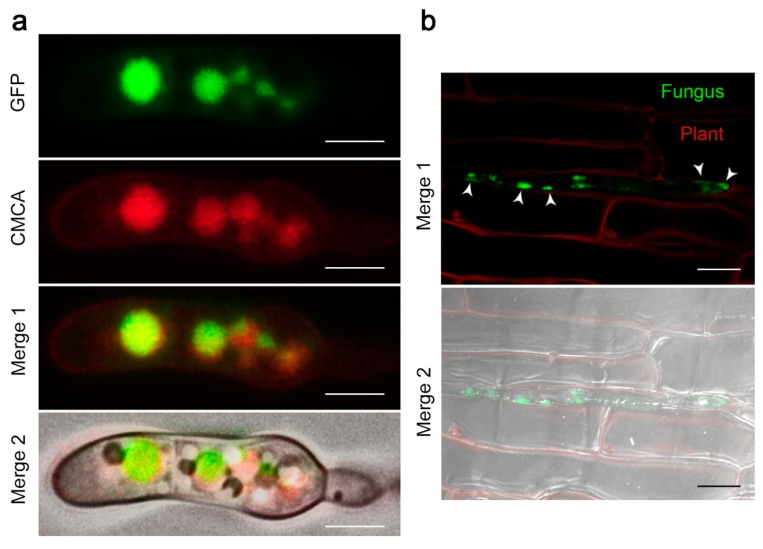
Subcellular localization of FocCP1. (**a**) Colocalization of vacuoles stained with CellTracker Blue (red) and FocCP1-GFP (green) in the conidia of ∆FocCP1-C-GFP strain. Bar = 5 µm. (**b**) Subcellular localization of FocCP1 during invasive growth was performed by placing conidial suspensions on banana leaves. Photographs were taken 24 h after incubation. Banana leaf cells were visualized by auto-fluorescence (red). Arrow heads point toward the secretion of FocCP1-GFP (green). Bar = 10 µm.

To determine the subcellular localization of FocCP1 in *Foc* TR4, conidia of the ∆FocCP1-C-GFP strain were stained with the vacuolar dye CellTracker Blue. Fluorescence microscopy analyses showed that FocCP1-GFP abundantly accumulated in vacuoles (Figure 4a). The protein was also observed to be expressed at different stages of conidial development (Figure S3). We also determined the expression of FocCP1 during plant invasion by inoculating the banana leaves with the ∆FocCP1-C-GFP strain and found that it was expressed during the tissue invasion and tended to be secreted to the surface of epidermal cells, confirming that FocCP1 plays a role in plant infection (Figure 4b). We further examined the subcellular localization of FocCP1 in planta through agroinfiltration of a FocCP1-GFP fusion construct. Transiently expressing the construct in the leaves of *Nicotiana benthamiana*, significant fluorescence of GFP was distributed mainly in the apoplast (Figure S4), which was in accordance with the results of an ApoplastP [26] prediction. Thus, our findings suggest that FocCP1 is carried by vacuoles in *Foc* TR4 and possibly then predominantly functions in the apoplast.

### 2.5. Recombinant FocCP1 Induces Necrosis in Banana Leaves

The *FocCP1* gene was inserted into the *pET32a* expression vector, subsequently transformed into *E. coli* and purified by Ni-NTA resin affinity. The His tag was removed by EK digestion. SDS-PAGE and Western blotting were performed and the results revealed that a purified protein with a molecular weight of 13 kDa was successfully obtained (Figure S5). 

**Figure 5:**
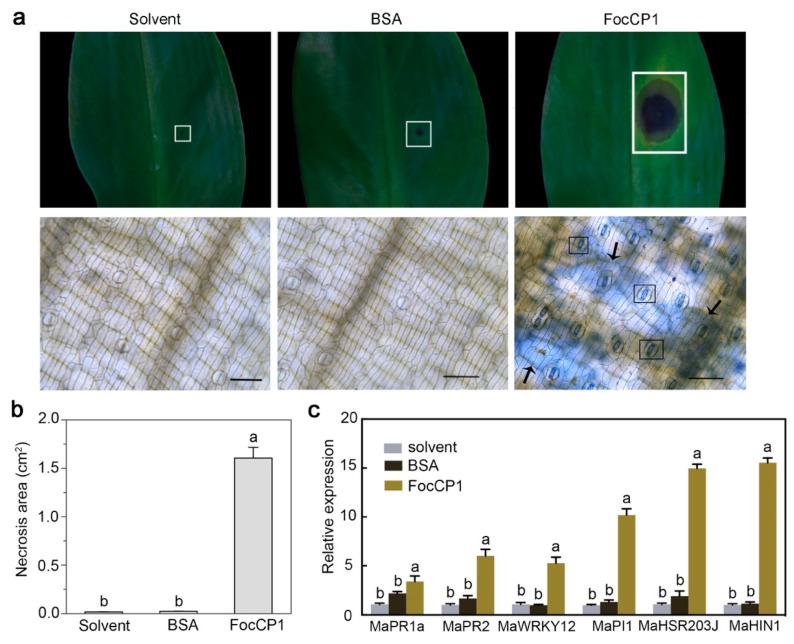
Biological function of FocCP1. (**a**) Banana leaves were treated with solvent, 50 µM BSA, or 50 µM FocCP1 protein for 48 h and cell death was observed using Trypan blue staining, the death of the stomata guard cells (black frames), and of epidermal cells (black arrows). Bars = 100 µm. (**b**) Areas of necrosis were measured on 8 leaves of 4 plants. Data are presented as means ± SDs and different letters above the columns indicate significant differences (*p* < 0.01 by Student’s *t*-test). (**c**) Relative expression of the immune marker genes *PR1a*, *PR2*, *WKRY12*, *PI1*, *HSR203J,* and *HIN1* in banana leaves treated with solvent (grey bars) or the FocCP1 protein (blue bars) at 6 hpi. *MusaActin* was used as an internal reference gene. Data are the means ± SDs from three independent experiments, and different letters above the columns indicate significant differences (*p* < 0.05 by Student’s *t*-test).

To verify the function of FocCP1 in causing necrosis, banana leaves were infiltrated with purified FocCP1 protein (50 µM), BSA (50 µM), or protein solvent (as controls). Infiltration of FocCP1 greatly induced lesion formation in the banana leaves, while no lesions were detected in the leaves treated with solvent or BSA (Figure 5a). For example, lesion areas produced by solvent and BSA were about 0.02 cm^2^, while lesion areas produced by FocCP1 were about 1.61 cm^2^ (Figure 5b). The leaves were stained with trypan blue to facilitate visualization of the occurrence of cell death (Figure 5a). By one day after inoculation, FocCP1-treated leaves displayed cell death around the inoculation site and in stomata guard cells and mesophyll cells, whereas solvent- and BSA-treated leaves were symptomless. We hypothesized that the early necrotic activity would be accompanied by increased accumulation of plant immune marker gene mRNA. To test this hypothesis, we performed qRT-PCR to detect whether FocCP1 had an effect on the expression of four genes, namely, the two pathogenesis-related genes *PR1a* and *PR2* [27], the transcription factor *WRKY12* [28], and protease inhibitor 1 (*PI1*) [29], as well as two cell death-related genes, *HSR203* and *HIN1* [30]. Banana leaves were infiltrated with FocCP1 protein, BSA, or solvent, and samples were collected 6 h post-inoculation (hpi), which was prior to the appearance of lesions. The expression of all six genes was higher in FocCP1-treated leaves than in the control leaves, especially for the two cell death-related genes, *HSR203* and *HIN1* (Figure 5c).

### 2.6. FocCP1 Contributes to the Pathogenicity of Foc TR4

**Figure 6:**
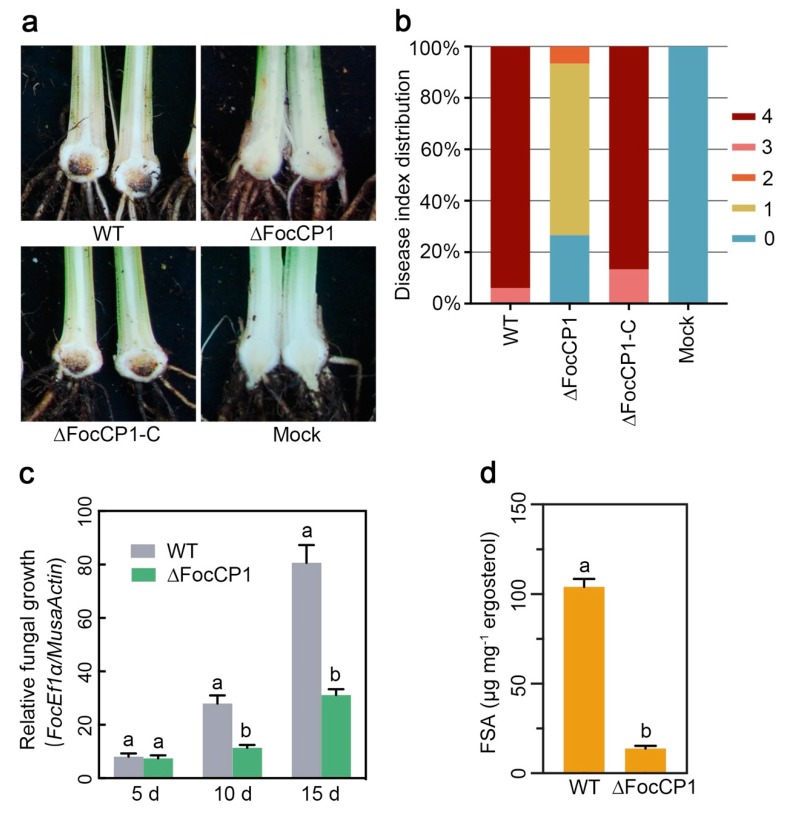
The deletion mutant ∆FocCP1 is attenuated in virulence in banana plantlets. (**a**) Disease phenotype and (**b**) disease index distribution in inoculated banana plantlets caused by the WT, ∆FocCP1, and ∆FocCP1-C-GFP strains at 5 weeks post-inoculation. (**c**) In planta fungal growth in banana roots of the inoculated plantlets. Relative fungal growth was evaluated by qRT-PCR analysis of the *FocEF1α* and banana *actin* genes and shown as ratios of *FocEF1α/MusaActin*. Data are the means ± SDs from three independent experiments and different the letters above the columns indicate a significant difference at the *p* < 0.01 level between the WT and ∆FocCP1 strains at the same time point. (**d**) The amount of fusaric acid (FSA) (mg^−1^ of fungal ergosterol) produced by the WT, ∆FocCP1, and ∆FocCP1-C-GFP strains in infected banana roots was determined after 10 days of inoculation. Data are the means ± SDs from three independent experiments and the different letters above the columns indicate a significant difference at *p* < 0.01 (Student’s *t*-test) between the WT and ∆FocCP1 strains at the same time point.

The virulence of ∆FocCP1 was evaluated on Cavendish banana plantlets by root infection assays. Compared with WT, the ∆FocCP1 strain showed a significant decrease in virulence to banana plants (Figure 6a), with only 66% percent and 27% percent of inoculated plantlets showing browning symptoms and no symptoms in leaves four weeks post-inoculation, respectively (Figure 6b). The complement strain ∆FocCP1-C-GFP showed recovered virulence and all of the inoculated plants died at four weeks post-inoculation. (Figure 6a,b). In addition, to examine whether targeted disruption of FocCP1 had an effect in planta on fungal growth, fungal biomass in the roots was qualified. Although the fungal biomass in the roots of the ∆FocCP1-inoculated banana plants increased gradually over 15 days post-inoculation (dpi), it showed a remarkable reduction compared to that of the WT-inoculated plants (Figure 6c). Since FSA is a key virulence factor in *Foc* TR4 [14], we assayed FSA production in banana roots inoculated with ∆FocCP1. As shown in Figure 6d, the FSA produced by ∆FocCP1 was below the threshold of LC-MS-MS detection. These data suggest that deletion of the *FocCP1* gene reduces the virulence of *Foc* TR4 on bananas and decreases infective growth within banana roots.

### 2.7. FocCP1 is Required for Successful Penetration of Banana Roots

**Figure 7:**
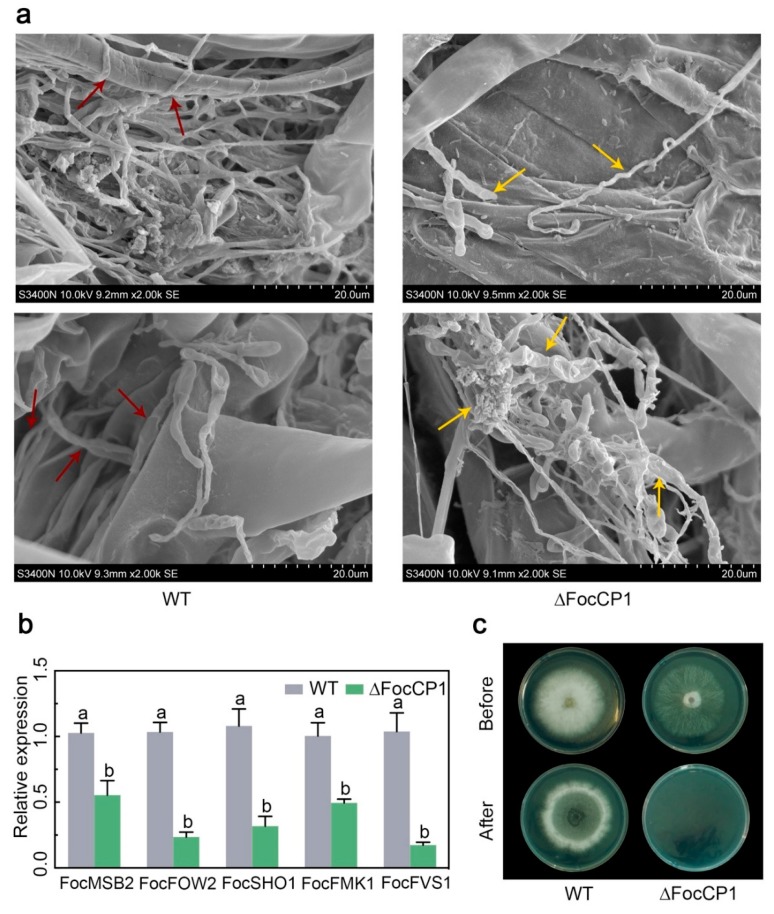
FocCP1 is required for successful penetration of *Foc* into banana roots. (**a**) Comparison of the infection behaviour of the WT and ∆FocCP1 strains on banana roots. Red arrows indicate penetration events and yellow arrows indicate unsuccessful penetration events. (**b**) Expression of infection-related genes of the WT and ∆FocCP1 strains. Data are the means ± SDs from three independent experiments. Different letters above the columns indicate significant differences at *p* < 0.01 (Student’s *t*-test) between the WT and ∆FocCP1 strains. (**c**) Comparison of the penetration ability against cellophane membranes. Fungal colonies were grown for 3 days on top of cellophane membranes placed on minimal medium (before). The cellophane membranes were then removed and the plates were incubated for an additional 2 days to examine hyphal growth.

As the ∆FocCP1 mutant grew well on the culture medium but not in the banana root, we investigated whether the deletion mutations affected the penetration process. To test this hypothesis, banana roots inoculated with conidia of the WT or ∆FocCP1 strains were examined 48 hpi using scanning electron microscopy (SEM). Most of the WT germinated conidia showed penetration events, whereas most of the ∆FocCP1 germinated conidia failed to penetrate and grew only on the root surface (Figure 7a). We further compared the expression levels of certain infection-related pathogenicity genes, namely, *FocMSB2*, *FocFOW2*, *FocSHO1*, *FocFMK1,* and *FocFVS1* [31,32,33,34,35], in the WT and ∆FocCP1 strains. qRT-PCR analysis showed that the expression levels of these pathogenicity-related genes in the ∆FocCP1 strain were significantly reduced in comparison to those in the WT strain (Figure 7b). Furthermore, cellophane penetration assays were performed to confirm the defect in penetration ability of the ∆FocCP1 strain. As shown in Figure 7c, the ∆FocCP1 strain showed a dramatically reduced ability to penetrate the cellophane membrane. These results indicate that FocCP1 is required for *Foc* TR4 to penetrate into banana roots, further confirming its contribution to the full virulence of *Foc* TR4.

## 3. Discussion

Fungal pathogens deliver hundreds of effectors into the host cell’s apoplastic space or directly into plant cells. Based on the published *Foc* II5 genome sequence, more than 500 effectors are predicted to be secreted into host tissue (data not published). To date, relatively few *Foc* effectors have been characterized as virulence factors. In the present study, FocCP1 (FOIG_10415), which was identified as a homologous protein of CP, was continuously expressed during the early invasion stage, indicating that it may play an important role in the early infection process (Figure 2). The other CP proteins were also implicated in fungal morphogenetic development; BcSpl1 from *B. cinerea*, MgSM1 from *M. grisea*, and VdCP1 from *Verticillium dahlia* were shown to be expressed at different fungal growth stages [19,20,36]. 

The positive role of CP proteins in the induction of plant defences against invading pathogens raises the question as to whether these proteins are effectors or elicitor molecules [37]. MoSm1 from *M. oryzae*, BcSpl1 from *B. cinerea*, and Sm1 from *T. virens* are considered to be PAMPs, since these CP proteins can be perceived by plant receptors and then trigger a plant immune response [38,39,40]. Some CP proteins also enhance the virulence of necrotrophic fungi, as many of them are expressed during –pathogen interactions and induce death of the affected tissue [24,41]. It was recently shown that FocCP1, which is identified in the secretome of *Foc*, triggered an immune response and caused systemic acquired resistance (SAR) in tobacco [42]. In our study, treating banana leaves with purified FocCP1 protein, acting as a virulence effector, induced cell death and upregulated expression of defence-related genes (Figure 5). Our results showed that necrosis was not observed in the infiltration area at concentrations below 20 µM, indicating that cell death caused by FocCP1 occurs in a dose-dependent manner. It was previously reported that low expression of SsCP1 did not result in obvious cell death in SsCP1-expressing transgenic *Arabidopsis thaliana*, whereas SsCP1 caused significant necrosis in the leaves of transiently expressing *N. benthamiana* [24]. The same results were also observed for VdCP1, a CP protein in *V. dahlia*, as the minimum concentration of VdCP1 required to cause the HR was 50 µM [36]. In addition, targeted disruption of FocCP1 significantly reduced virulence and its aerial growth was severely impacted compared to that of the WT strain (Figure 3). When fungal hyphae emerge from aqueous growth medium to form aerial hyphae and produce conidia, these growth structures are covered with a layer of hydrophobins that render them hydrophobic [37]. It has been reported that CP proteins are homologous to hydrophobins [16], which facilitate hyphal growth, adhesion, host-cell penetration, and sporulation [43]. The CP proteins also aggregate in a similar manner with hydrophobins [44]. This behaviour suggested that FocCP1 may have a function similar to that of hydrophobins. The CP-like protein MSP1′s activity in virulence was shown to be like the hydrophobin MHP1 in *M. grisea*. Nevertheless, no differences between WT and most CP protein-knockout strains, such as FgCPPs from *Fusarium graminearum* and EPL1, were detected with respect to conidiation, aerial growth, and hydrophobicity [41,45]. In *BcSpl1*-knockout mutants, wettability of the fungal colony surface, which was tested because hydrophobin mutants usually show easier entrance of water to the interior of the colony, showed no difference compared to that of wild type stain [19]. Possibly, with respect to hydrophobicity, there is considerable differentiation among CP proteins [19,41,44,45] and the hydrophobic characteristic of FocCP1 is not a general property that can be found in all members of the CP protein family. Biochemical and structural analyses of FocCP1 are necessary to further elucidate its specific hydrophobic role in the pathogenicity of *Foc* TR4.

The ∆FocCP1 mutant showed no significant differences from the WT strain in aqueous growth; however, the fungal biomass was significantly reduced in ∆FocCP1-inoculated banana plantlets and the virulence of the ∆FocCP1 strain was considerably attenuated compared to that of the WT (Figure 6c). The growth defect in planta rather than in vitro in broth culture suggested that the disruption of FocCP1 affected *Foc* TR4 penetration ability, indicating that FocCP1 is also important for invasive hyphal growth in banana roots. SEM observations of the inoculated roots showed that the ∆FocCP1 strain was defective in its ability to penetrate the root tissue (Figure 7a), which is correlated with the decreased expression levels of several infection-related genes, including *FocMSB2*, *FocFOW2*, *FocSHO1*, *FocFMK1*, and *FocFVS1* [31,32,33,35]. These infection-related genes were previously shown to play important roles in the penetration process of *F. oxysporum*. FSA produced by *Foc* TR4 was shown to be involved in causing the wilt symptom and was essential for the virulence on banana [14,15]. Here, we observed that the ∆FocCP1 strain produced significantly less FSA than the WT strain (Figure 6d). Since the effectors and all the other virulence factors secreted by pathogenic fungi coordinate together during plant infection and localization within host tissues [46] and the role of FocCP1 in FSA biosynthesis differs from the FSA biosynthetic gene (FUB) cluster, it is likely that an unidentified downstream virulence factor of FocCP1 regulates FSA biosynthesis in *Foc* TR4.

We have shown that FocCP1 is responsible for the infectivity of *Foc* TR4. One possible reason for this is that the necrosis-inducing activity of FocCP1 generates dead tissue appropriate for the growth of *Foc* TR4. An alternative, but not contradictory, explanation may be that FocCP1 acts via its putative hydrophobin-like activity and therefore contributes to virulence by facilitating plant invasion by *Foc* TR4. Since *Foc* TR4 is a hemibiotrophic pathogen, there is an early asymptomatic biotrophic phase and a late necrotrophic stage that is characterized by tissue degradation and disease symptoms [47]; however, the mechanism behind the transition between the two phases remains unknown. We hypothesized that *Foc* TR4 might secrete some effector proteins to suppress banana defence responses and begin to induce associated cell death to accelerate the transition. FocCP1 was suggested to play an important role in mediating the transition from biotrophy to necrotrophy in hemibiotrophic plant pathogens.

## 4. Materials and Methods 

### 4.1. Fungal Strains, Plants, and Growth Conditions

Wild type *Fusarium oxysporum* f. sp. *cubense* TR4 strain II5 (NRRL#54006), designated as VCG 01213, was used in this study [48]. The FocCP1 gene deletion mutant was derived from this isolate. The wild-type strain and transformants generated in this study were grown at 28 °C on PDA for mycelial growth tests. MBL broth was used for conidiation assays [49].

### 4.2. Bioinformatics Analysis

Whole-genome protein sequences of 22 genomes, including 21 from *Fusarium* spp. and 1 from *Trichoderma atroviride* as the outgroup, were downloaded from the GenBank assembly database and the assembly accession numbers are listed in Table S1. BLASTp was performed using the FOIG_10415 (designation from the Fusarium Comparative Database at the Broad Institute) protein sequence as a query to search the homologues in the NCBI GenBank database. Then, FOIG_10415, which was designated as FocCP1 protein, was used to search for its homologues in all the downloaded protein sequences using BLASTp. ClustalX 2.1 was used to generate a multiple alignment from the protein sequences [50], which was turned into a graph with ESPrint 3.0 [51]. Secondary structure information on the alignment graph was drawn in ESPrint 3.0 from the Protein Data Bank (PDB) 3D structure of the protein P81702. A protein evolution model test was carried out by Prottest 3.4.2 on the CP family [52] and the WAG + G model was selected as the best model either via the Akaike information criterion (AIC) or the Bayesian information criterion (BIC). Then, for phylogenetic analysis, the maximum likelihood (ML) method was carried out using the best fit model by PhyML 3.1 with 1000 bootstrap tests [53]. SignalP 4.0 was used for signal peptide prediction. Sequence motifs were identified and analyzed with the online web server provided by the MEME suite [54].

### 4.3. Cloning, Expression, and Purification of the FocCP1 Protein

A 366 bp FocCP1 fragment without the predicted signal peptide and stop codon was inserted into a pET32a vector using the specific primers presented in Table S2 [55]. The recombinant plasmid pET32a-FocCP1-His-tag or empty plasmid pET32a was transformed into *E. coli* BL21 cells. Colonies were selected on LB solid medium with 50 µg/mL ampicillin (Sigma, St. Louis, MO, USA).

*E. coli* BL21 colonies were transferred into LB medium with ampicillin and grown overnight. Then, 1 mL of the suspension was added to 200 mL of LB medium with ampicillin at 37 °C and cultured to an OD_600_ of 0.6, after which 0.5 mM isopropyl-β-thiogalactopyranoside (IPTG) was added at 20 °C while shaking overnight to induce expression of the recombinant protein. Cells were collected and lyzed by sonication and the supernatant after centrifugation was applied to a Ni-NTA column equilibrated with binding buffer (8 M urea, 50 mM Tris, 300 mM NaCl, pH 8.0). After a washing step, proteins were eluted with elution buffer (8 M urea, 50 mM Tris, 300 mM NaCl, 500 mM imidazole, pH 8.0) and then dialyzed in PBS overnight. Fractions containing the FocCP1 proteins were digested with EK overnight at 4 °C, then the digested fraction was further applied to a Ni-NTA column as described above, without urea in the buffers. Th recombinant protein FocCP1-His and purified FocCP1 were analyzed by sodium dodecyl sulfate-polyacrylamide gel electrophoresis (SDS-PAGE) and immunoblotting (anti-His antibody, 1:500, Sangon Biotech, Shanghai, China, D110002; goat anti-rabbit antibody, 1:8000, Sangon Biotech, D110058).

### 4.4. Histochemical Assays

The necrosis-inducting activity, trypan blue staining, and defence-related gene expression were assayed in FocCP1-infiltrated banana leaves. Solvent/BSA-infiltrated banana leaves were used as controls. Infiltration tests were carried out with detached banana (cultivar Brazilian) leaves with the aid of a needle used to poke holes in the leaves. Pieces of round filter paper of 5 mm radius with 10 µL droplets of FocCP1, solvent, or BSA were attached to the banana leaves. The infiltrated banana leaves were incubated at 28 °C under conditions of high humidity on water-soaked filter paper in a closed container.

Trypan blue staining was performed to visualize cell death, as previously described [56]. The leaves were boiled for 5 min in a 1:1 mixture of ethanol and 0.33 mg/mL trypan blue in lactophenol and destained overnight in 2.5 g/mL chloral hydrate in water. The stained leaves were examined under a Leica DM4B imaging system (Leica Microsystems, Mannheim, Germany). The experiments were repeated at least three times with similar results.

### 4.5. RNA Extraction and qRT-PCR Analysis

Total RNA was extracted from hyphae or banana roots using an RNA out kit (Tiandz, Beijing, China), following the manufacturer’s instructions. Genomic DNA was eliminated by RNase-free Recombinant DNase I (Takara, Kusatsu, Japan). The first-strand cDNA was synthesized by AMV Reverse Transcriptase (Takara, Japan) and quantitative real-time reverse transcriptase PCR (RT-PCR) was carried out in a StepOne real-time PCR system (Applied Biosystems, Carlsbad, CA, USA) with the Fast SYBR Green Master Mix (Applied Biosystems). *FocEF1α* was used as the endogenous reference gene and the relative expression of each gene under different conditions was determined using the 2^-∆∆CT^ method. Expression of all the samples was examined in three biological replications with two technical duplicates each. All the primer pairs used are listed in Table S2.

### 4.6. Construction of A Gene-Deletion Mutant and Complementary Strain

FocCP1-targeted disruption was constructed using the protocol described previously [57]. Briefly, the *FocCP1* gene was replaced with a hygromycin-resistance cassette (*HPH*) driven by a constitutive trpC promoter, which was amplified from the PBS-HPH1 vector [58]; subsequent putative deletion mutants were identified through PCR assays, with the primer pairs listed in Table S2. For construction of the FocCP1-GFP fusion cassette, FocCP1 was amplified with the primer pair FocCP1-COM-F/R (Table S2). The FocCP1-GFP fusion vector was then transformed into the ∆FocCP1 strain for complementation and localization. Transformants were selected with geneticin and further examined via PCR with primer pair FocCP1-COM-ID-F/R (Table S2).

### 4.7. Pathogenicity Tests and Fungal Biomass Estimation

Cavendish banana variety Brazilian (*Musa* spp. AAA group) plantlets with six to seven leaves were inoculated with *Foc* strains at a concentration of 1000 conidia/g of soil. For the pathogenicity test, the disease severity of each tested plant was evaluated. The disease index range was as follows: 0 (no symptoms), 1 (some brown spots in the inner rhizome), 2 (less than 25% of the inner rhizome showed browning), 3 (up to 3/4 of the inner rhizome showed browning), and 4 (entire inner rhizome and pseudostem were dark brown, dead). In addition, the penetration behaviour of the strains was examined on cellophane membranes using a previously described protocol [49].

To estimate and compare the fungal biomass inside the plant, banana roots were collected at 5, 10, and 15 dpi and the transcript levels of the *FocEF1α* and banana *MusaActin* genes were determined by qRT-PCR using a pair of *FocEF1α*-specific primers and a pair of banana *MusaActin*-specific primers (Table S2). The relative fungal biomass was calculated by normalizing *FocEF1α* to banana *MusaActin*, shown as the ratio of *FocEF1α/MusaActin* [49].

### 4.8. Analysis of FSA

The FSA content was determined via LC-MS/MS analysis as described previously [14]. The amount of ergosterol was used as the internal control in each *Foc* sample incubated on PDA plates. The ergosterol was extracted and analyzed as previously described [59].

### 4.9. Scanning Electron Microscopy Observation

For SEM observation, Brazilian (*Musa* spp. AAA group) plantlets with six to seven leaves were inoculated by dipping the roots into a *Foc* spore suspension (1 × 10^7^ spores/L) on a rotary shaker at 80 rpm and root samples were collected 48 h later. The root samples were treated as described previously [49] and observed using a Hitachi Model S-3400N scanning electron microscope (Hitachi, Tokyo, Japan).

### 4.10. Subcellular Localization Assays and Fluorescence Microscopy

The FocCP1 coding region was amplified using the primers FocCP1-SL-F/R (Table S2), digested with *Kpn*I/*Xba*I, and cloned into pCAMBIA1301-eGFP [60]. *Agribacterium tumefaciens* strain GV3101 carrying the recombinant plasmid pCAMBIA1301-eGFP-FocCP1 or the empty vector pCAMBIA1301-eGFP was cultured to OD_600_ 0.5 and then incubated at room temperature (RT) for 3 h before being infiltrated into *Nicotiana benthamiana* leaves. GFP fluorescence was observed with a laser confocal microscope (LSM 710, Carl Zeiss, Oberkochen, Germany) 3 days post-infiltration. For fluorescence microscopy of the ∆FocCP1-C-GFP strain, vacuolar staining was performed using CellTracker Blue CMCA (blue) (Invitrogen, Camarillo, CA, USA) at a final concentration of 100 µM and incubation with conidia or hyphae for 15 min. The conidia or hyphae were also stained with a nucleus tracker DAPI (4′, 6-diamidino-2-phenylindole) (Invitrogen, USA) at a final concentration of 300 nM. Samples were visualized under a Leica DM4B imaging system (Leica Microsystems, Germany).

## Supplementary Materials

Supplementary materials can be found at https://www.mdpi.com/1422-0067/20/15/3785/s1.

## Abbreviations

*Foc* TR4	*Fusarium oxysporum* f. sp. *cubense* tropical race 4
CP	cerato-platanin
FocCP1	The first cerato-platanin protein from *Foc* TR4
PAMPs	pathogen-associated molecular patterns
HR	hypersensitive response
PTI	linear dichroism
ETI	effector-triggered immunity
SIX	secreted in xylem
SSCRP	small secreted cysteine-rich proteins
