# Supplementary figures and images for: A Cerato-Platanin Family Protein FocCP1 Is Essential for the Penetration and Virulence of Fusarium oxysporum f. sp. cubense Tropical Race 4
Source: Int J Mol Sci. 2019 Aug 2;20(15):3785. doi: 10.3390/ijms20153785 (PMC6695778; doi:10.3390/ijms20153785)

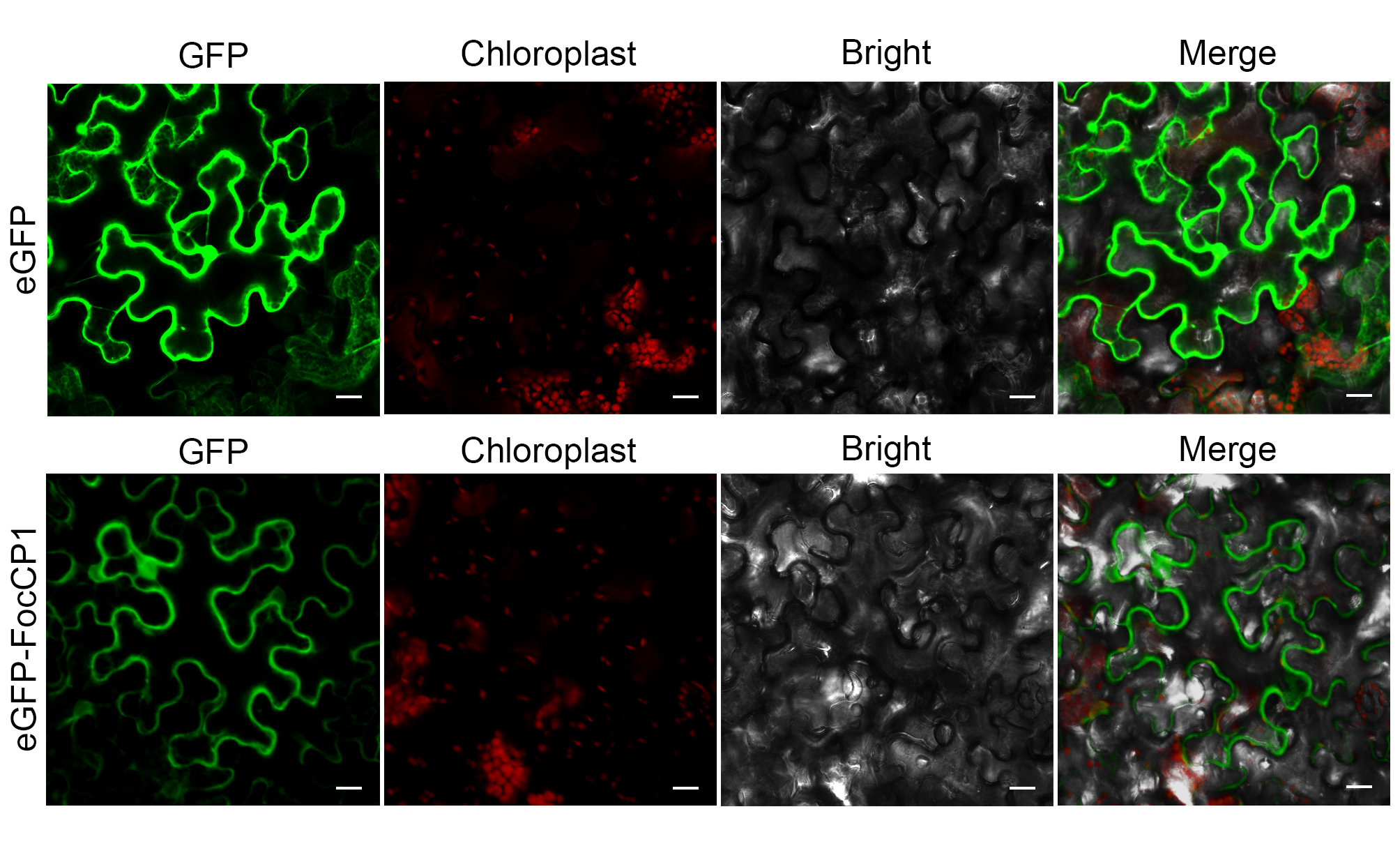

Supplement: Supplementary file 1 [file ijms-20-03785-s001.zip › Fig. S4.jpg]

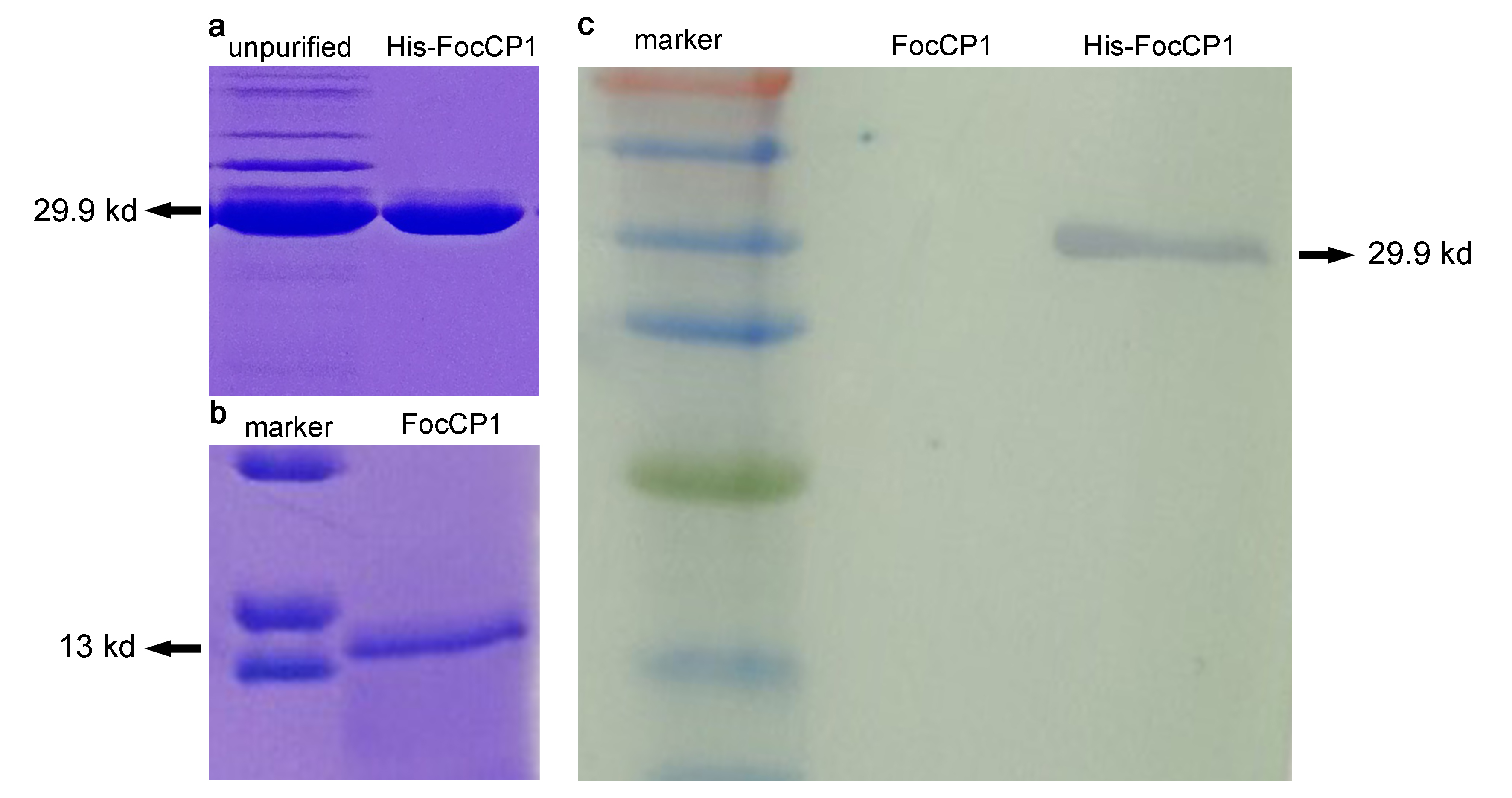

Supplement: Supplementary file 1 [file ijms-20-03785-s001.zip › Fig. S5.jpg]

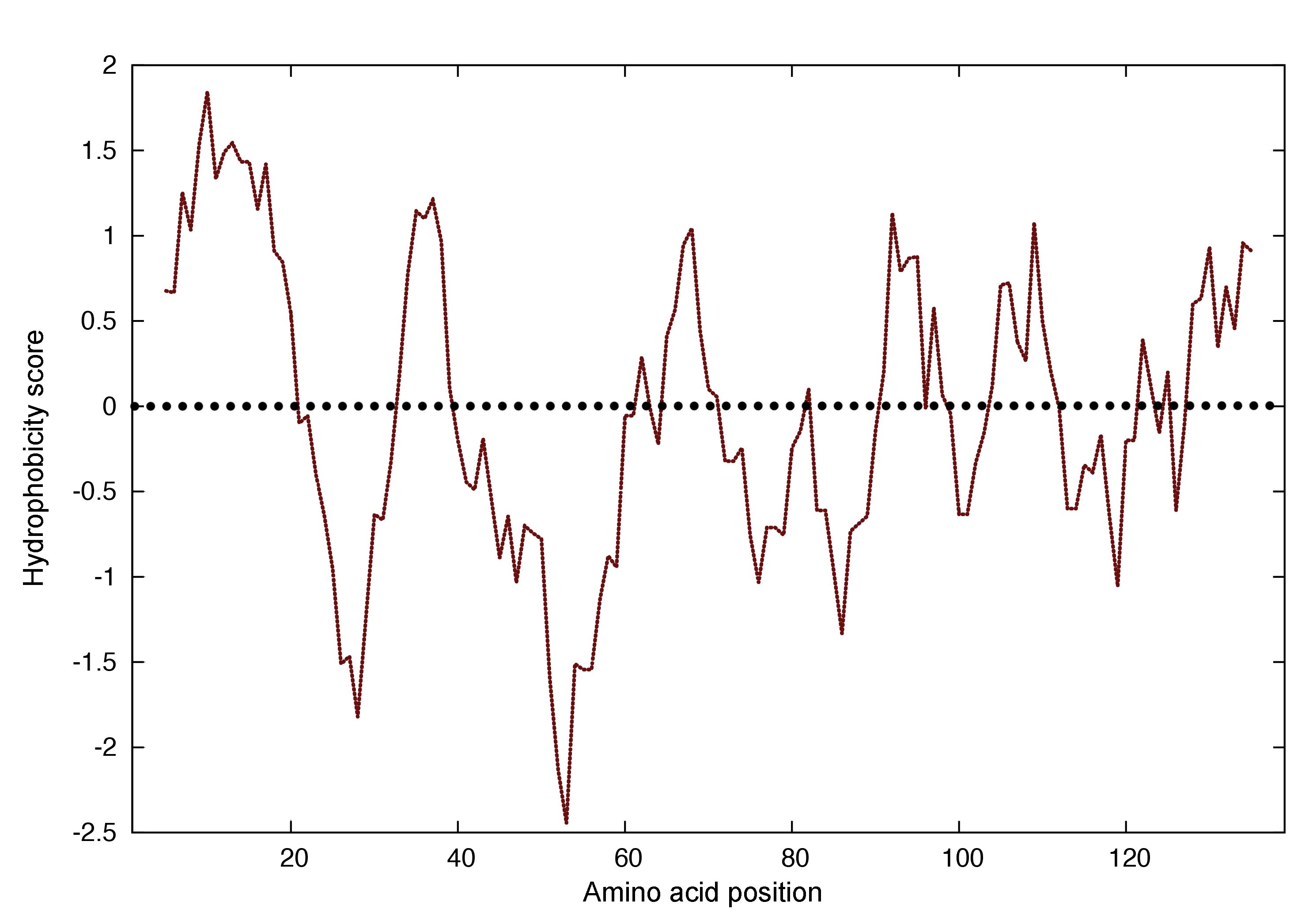

Supplement: Supplementary file 1 [file ijms-20-03785-s001.zip › Fig. S1.jpg]

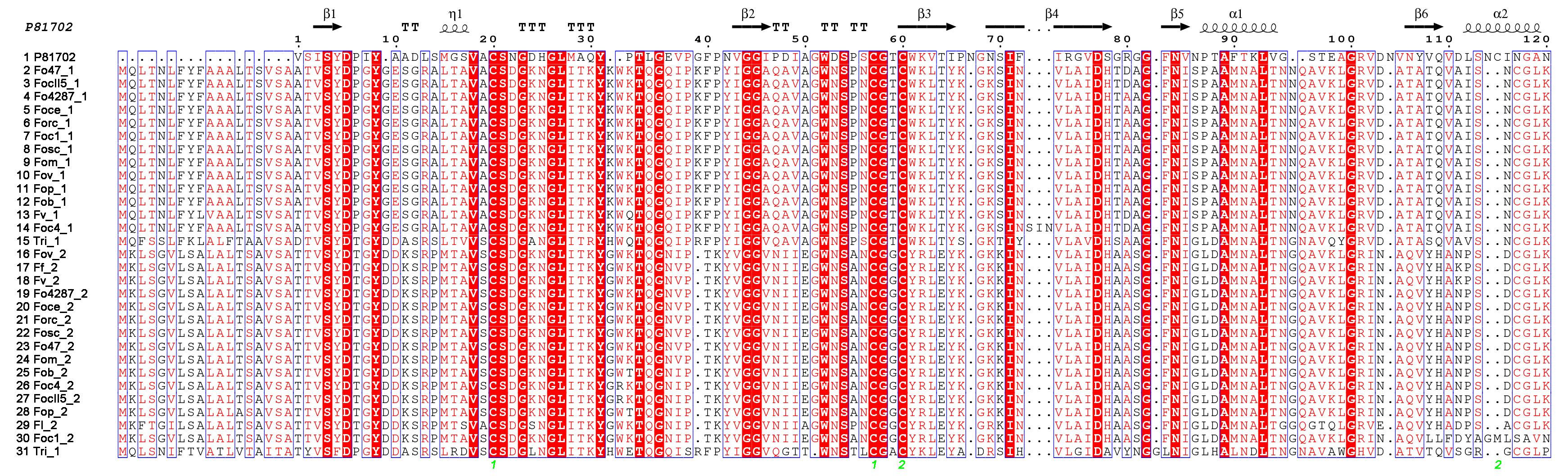

Supplement: Supplementary file 1 [file ijms-20-03785-s001.zip › Fig. S2.jpg]

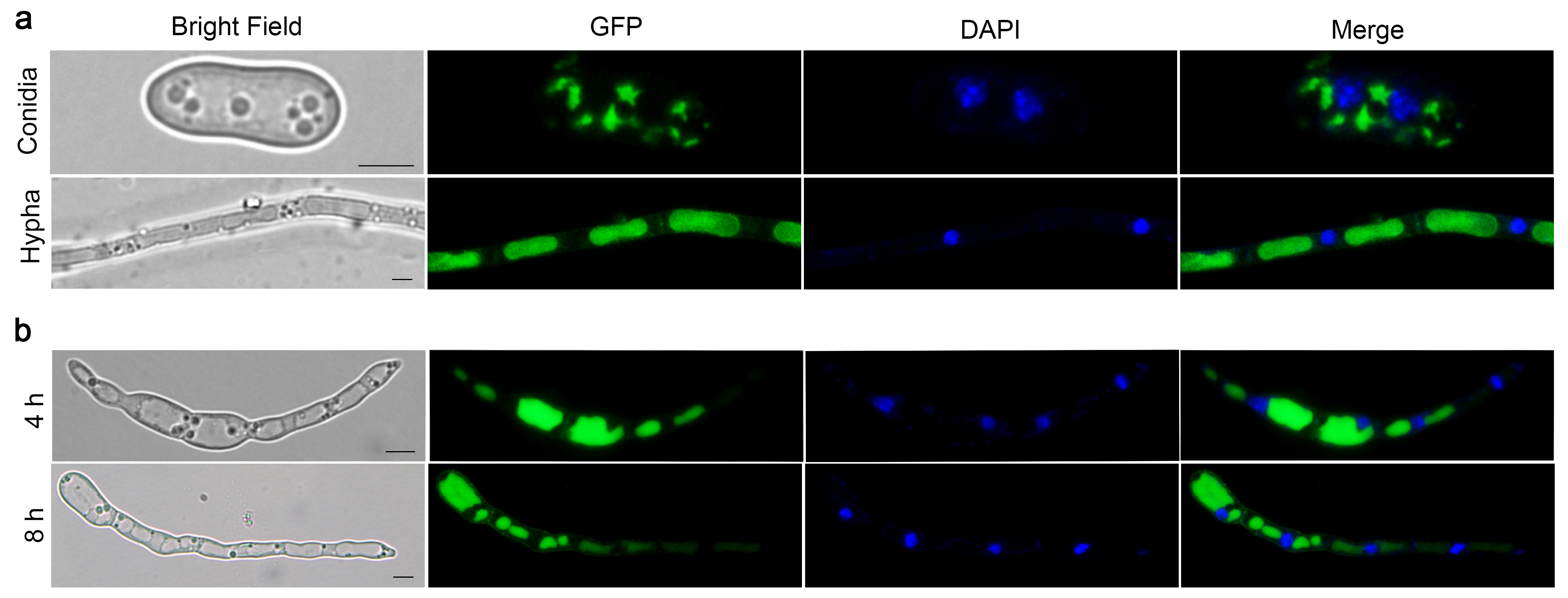

Supplement: Supplementary file 1 [file ijms-20-03785-s001.zip › Fig. S3.jpg]
